# Meta-Analysis of Hepatic Arterial Infusion for Liver Metastases From Colorectal Cancer

**DOI:** 10.3389/fonc.2021.628558

**Published:** 2021-03-10

**Authors:** Yan Zhang, Kaili Wang, Tao Yang, Yibo Cao, Wanling Liang, Xiangdong Yang, Tianbao Xiao

**Affiliations:** ^1^The Second Clinical School, Guangzhou University of Traditional Chinese Medicine, Guangzhou, China; ^2^China Academy of Chinese Medical Sciences, Beijing, China; ^3^College of Clinical Medicine, Chengdu University of Traditional Chinese Medicine, Chengdu, China; ^4^The First Affiliated Hospital of Guizhou University of Traditional Chinese Medicine, Guiyang, China; ^5^Colorectal and Anal Surgery, Chengdu Anorectal Hospital, Chengdu, China

**Keywords:** colorectal cancer, liver, metastases, meta-analysis, hepatic arterial infusion chemotherapy

## Abstract

The aim of the present study was to evaluate the potential benefits of hepatic arterial infusion chemotherapy (HAIC) in the management of colorectal liver metastases (CRLM). Electronic databases, including PubMed, EMBASE, Medline, Web of Science, and Cochrane Library, were comprehensively searched from inception to November 2020. Prospective randomized trials with HAIC vs. systemic chemotherapy (SC) were selected. The overall survival (OS), tumor response rates (RRs), progression-free survival (PFS), and corresponding 95% confidence intervals (CIs) were assessed in the meta-analysis. Subsequently, the heterogeneity between studies, sensitivity, publication bias, and meta-regression analyses were performed. Finally, 18 studies, which contained 1,766 participants (922 in the HAIC group and 844 in the SC group) were included. There was a significantly higher OS rate in the HAIC as palliative treatment group (HR, 0.17; 95% CI, 0.08–0.26; *P* = 0.000) and HAIC as adjuvant treatment group compared with SC group (HR, 0.63; 95% CI, 0.38–0.87; *P* = 0.000). The complete and partial tumor RRs were also increased significantly in the HAIC as palliative treatment group (RR = 2.09; 95% CI, 1.36–3.22; *P* = 0.001) and as adjuvant treatment group compared with SC group (RR = 2.14; 95% CI, 1.40–3.26; *P* = 0.000). However, PFS did not differ significantly between the HAIC and SC groups (*P* > 0.05). Meta-regression analysis showed potential covariates did not influence on the association between HAIC and OS outcomes (*P* > 0.05). The results of the present study suggested that HAIC may be a potential therapeutic regimen that may improve the outcomes of patients with CRLM. The present meta-analysis has been registered in PROSPERO (no. CRD 42019145719).

## Introduction

**Figure 1:**
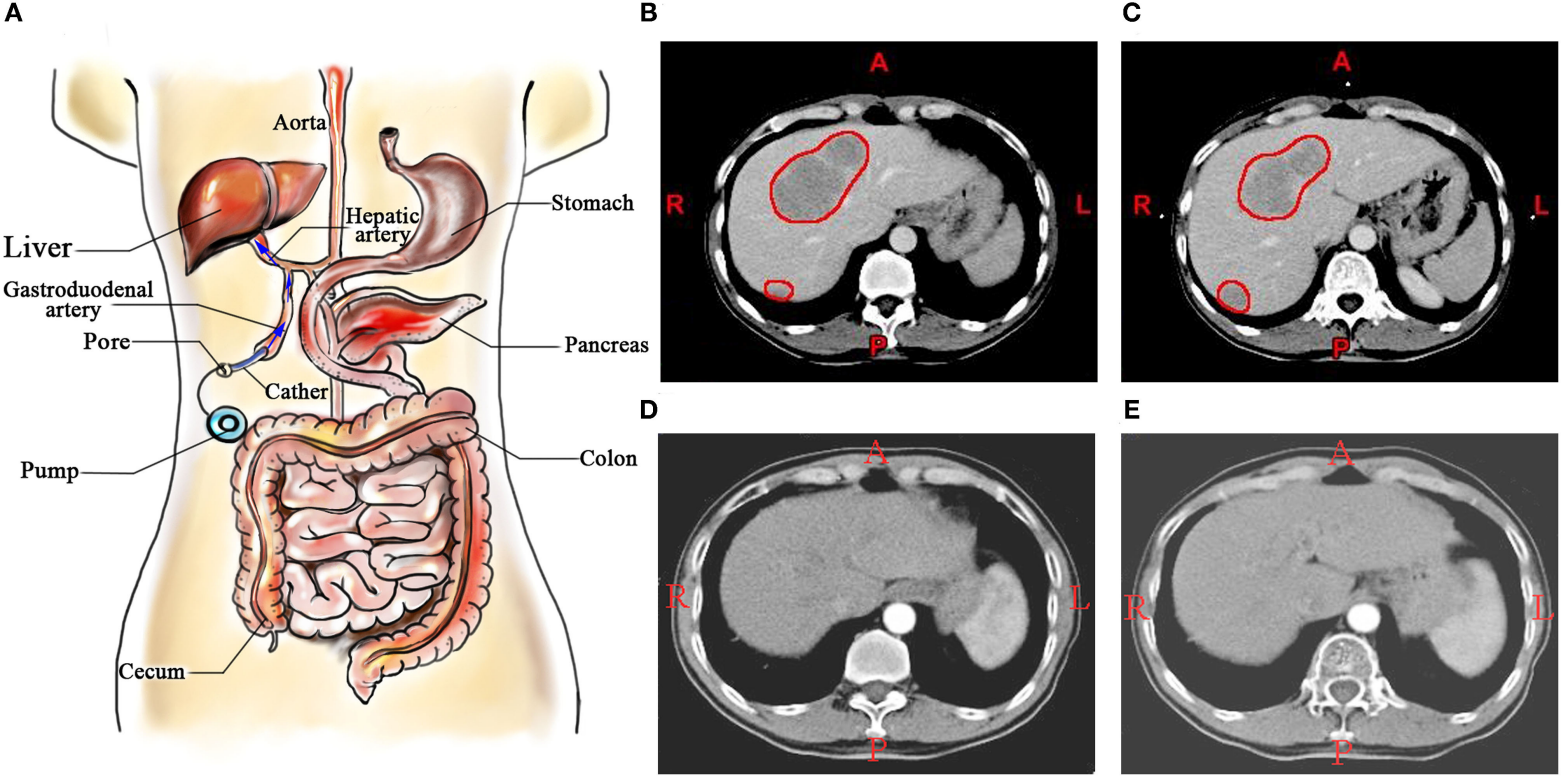
**(A)** Schematic diagram of HAIC; **(B,C)** A patient with colorectal liver metastases, and the metastatic lesions in liver were marked by red circles; **(D,E)** The patient treated by HAIC for 24 months. HAIC, hepatic arterial infusion chemotherapy.

Colorectal cancer (CRC) is the third most common type of cancer in terms of incidence (10.2%) and the second leading cause of cancer-associated death (9.2%). In 2018, there were over 1.8 million new CRC cases and 881,000 estimated deaths worldwide (1). It is estimated that there were 51,020 deaths in 2019 in the USA (2). The liver is the most frequent site of distant metastases of CRC (3), and serves as the leading cause of death in patients with CRC. It is estimated that 50% of patients develop liver metastases, of which ~25% of patients present with synchronous metastases and another ~50% with developing metachronous metastases (4). R0/R1 resection of both the liver metastases and the primary CRC has been demonstrated to improve long-term survival times to a certain degree (5, 6). Of patients with CRC with liver metastases, 15–20% of patients with liver metastases undergo surgical operation at presentation (7, 8), and the 5-year overall survival (OS) rates are in the range of 34–36% (3, 4). Regarding unresectable colorectal liver metastases (CRLM), therapeutic management is more controversial, and is generally associated with less favorable prognoses (5). Thus, optimization of the treatment for CRLM is required. Over the past decade, effective systemic and regional chemotherapy for CRLM has been introduced. Hepatic arterial infusion chemotherapy (HAIC), a locoregional therapy for treatment with liver metastases, is a potentially appealing treatment and it has been developed over the last three decades for patients with CRLM (9). HAIC possesses theoretical advantages over standard intravenous systemic chemotherapy (SC) due to the anatomical characteristics. Portal vein to parenchyma, while hepatic artery to metastatic tumor of the liver make it possible to cure patients with CRLM (6, 7). The schematic diagram of HAIC is presented in Figure 1.

In several randomized controlled trials (RCTs), floxuridine (FUDR)-based HAIC has reproducibly yielded higher tumor response rates (RRs) compared with SC (10–15). However, OS outcomes in all the RCTs referenced above have not improved. According to three previous meta-analyses published >10 years ago (16–18), HAIC did not significantly improve survival compared with SC. A total of 18 RCTs comparing HAIC with SC based on multiple chemotherapy drugs (such as irinotecan, leucovorin, oxaliplatin, and regorafenib) have been performed (10–15, 19–30), and have produced contradicting results. The survival benefits of HAIC, which is contested, should be re-examined in the era of multidisciplinary team strategies, and compared with the combination of drugs for treatment of patients with CRLM. Therefore, in the present study, a meta-analysis was performed to collectively quantitatively analyze previous clinical studies.

## Materials and Methods

### Protocol Registration

The present study was previously registered in PROSPERO during Nov 2019 (registration no. CRD 42019145719; crd.york.ac.uk/PROSPERO).

### Eligibility Criteria

This study developed the inclusion and exclusion criteria based on “PICOS” principles. Inclusion criteria were as follows: (i) Design of studies, prospective RCTs; (ii) patients (P), patients with CRLM, which is defined as ≥4 metastases or metastatic nodules >50 mm, bilobar characteristics, invasion of pedicle lymph nodes, serum levels of carcinoembryonic antigen >200 ng/ml; (iii) intervention (I), HAIC; (iv) control (C), SC; (v) outcomes (O), the primary endpoints were OS, which was defined as the time from identification to death by any cause. The secondary endpoint was RRs, which was defined as the percentage of complete (tumor disappearance), or partial (tumor shrinkage ≥50%) RRs, and progression-free survival (PFS), which was defined as the length of time that patients lived with the tumor without evidence of progression of the cancer.

The exclusion criteria were: (i) Irrelevant studies and duplicate literature; (ii) studies without useful data; and (iii) letters, reviews, case reports, comments, laboratory studies, and meta-analyses.

### Search Methodology

The selection and systematic review of clinical studies were performed and reported in accordance with the Preferred Reporting Items for Systematic Reviews and Meta-Analyses (PRISMA) statement (8). The search was limited to RCTs published in English. Electronic databases including PubMed, EMBASE, Medline, Web of Science, and Cochrane Library were comprehensively searched from inception to November 2020. The following search terms were searched using combinations of medical subject headings terms and the following free words: Colon/Rectal, colorectal/cancer/tumor, carcinoma/neoplasm/liver/hepatic and metastases/hepatic arterial infusion/trans-arterial chemoembolization/chemotherapy. In addition, potentially relevant references were also obtained. Using the PubMed database as an example, the search strategy used was as follows: (i) colon OR rectal OR colorectal; (ii) cancer OR tumor OR carcinoma OR neoplasm; (iii) colon OR rectal OR colorectal AND cancer OR tumor OR carcinoma OR neoplasm; (iv) liver or hepatic or metastases; (v) colon OR rectal OR colorectal AND cancer OR tumor OR carcinoma OR neoplasm AND liver OR hepatic OR metastases; (vi) hepatic arterial infusion OR trans-arterial chemoembolization OR HAIC OR TACE OR chemotherapy; and (vii) colon OR rectal OR colorectal AND cancer OR tumor OR carcinoma OR neoplasm AND liver OR hepatic OR metastases AND hepatic arterial infusion OR trans-arterial chemoembolization OR HAIC OR TACE OR chemotherapy; where TACE stands for trans-arterial chemoembolization.

### Study Selection

All search results were combined in Endnote™, Version X8 (Thompson Reuters). Duplicates were removed manually. Two investigators independently screened the studies based on the titles and abstracts. If the article met the eligibility criteria, the full text was read. Any discrepancies between the two investigators were resolved by discussion or third-party consensus.

### Data Extraction

Two investigators used the inclusion and exclusion criteria to retrieve relevant citations. Using a standardized data extraction form, two investigators independently extracted the following data from each study: (i) Study ID, including the name of the first author and publication year; (ii) country where the study was performed; (iii) study subjects, number of participants and their ages; (iv) treatment regimens for the treatment and control groups; and (v) the primary endpoint (OS) and the secondary endpoints (RRs and PFS). For reports of the same trial at different follow-up periods, data from the last report were used for analysis. If insufficient details were reported, the authors were contacted for further information. Any disagreements were resolved by consensus.

### Quality Assessment

The Cochrane Collaboration tools for assessing risk of bias and the Jadad's scale (31) were both used to evaluate the quality of the included RCTs. Jadad's scale with a maximum of five scores assesses the quality of the study based on three criteria: (i) Randomization; (ii) double blinding; and (iii) withdrawals and dropouts. A study was awarded a maximum of 2 points for randomization, 2 points for double blinding, and 1 point for withdrawals and dropouts. A final score ≥3 or above was regarded as high quality, whilst a score of 0–2 was considered low quality. Any disagreements during assessment were resolved by consensus.

### Statistical Analysis

All data were analyzed using Stata version 13.0 (Stata Corporation). Heterogeneity amongst studies was assessed using a Q test and an I^2^ test before determining the pooled effect (32). A fixed effects model and a random effects model were based on the results of the Q test and I^2^ test. A fixed effects model was adopted if I^2^ < 50% and *P* > 0.1. Otherwise, a random effects model was used. For the outcomes, OS and PFS, which were time-to-event variables, were expressed as pooled hazard ratios (HR). The HR of OS and PFS with 95% confidence intervals (CIs) were directly extracted from the Kaplan-Meier survival curves or calculated using a calculation sheet as described by Tierney et al. (33). The logarithm of HRs and the corresponding standard error (SE) were applied as data points for the meta-analysis. The tumor RRs, which was dichotomous data, expressed as pooled relative risk (RR) and 95% CIs were calculated. The significance of pooled effects was determined using a Z test; *P* < 0.05 was considered to indicate a statistically significant difference. Possible sources of heterogeneity were assessed performing meta-regression to evaluate the impact of covariates on overall heterogeneity, the restricted maximum likelihood (REML) estimation method proposed by Harbord et al. (9) was used for meta-regression. Sensitivity analysis was utilized to investigate the influence of a high-risk study on overall meta-analysis. Possible publication bias was determined using Egger's regression asymmetry test (34). Additionally, a contour-enhanced funnel plot was used to distinguish the detailed reasons underlying publication bias (35).

## Results

### Study Selection Outcome

**Figure 2:**
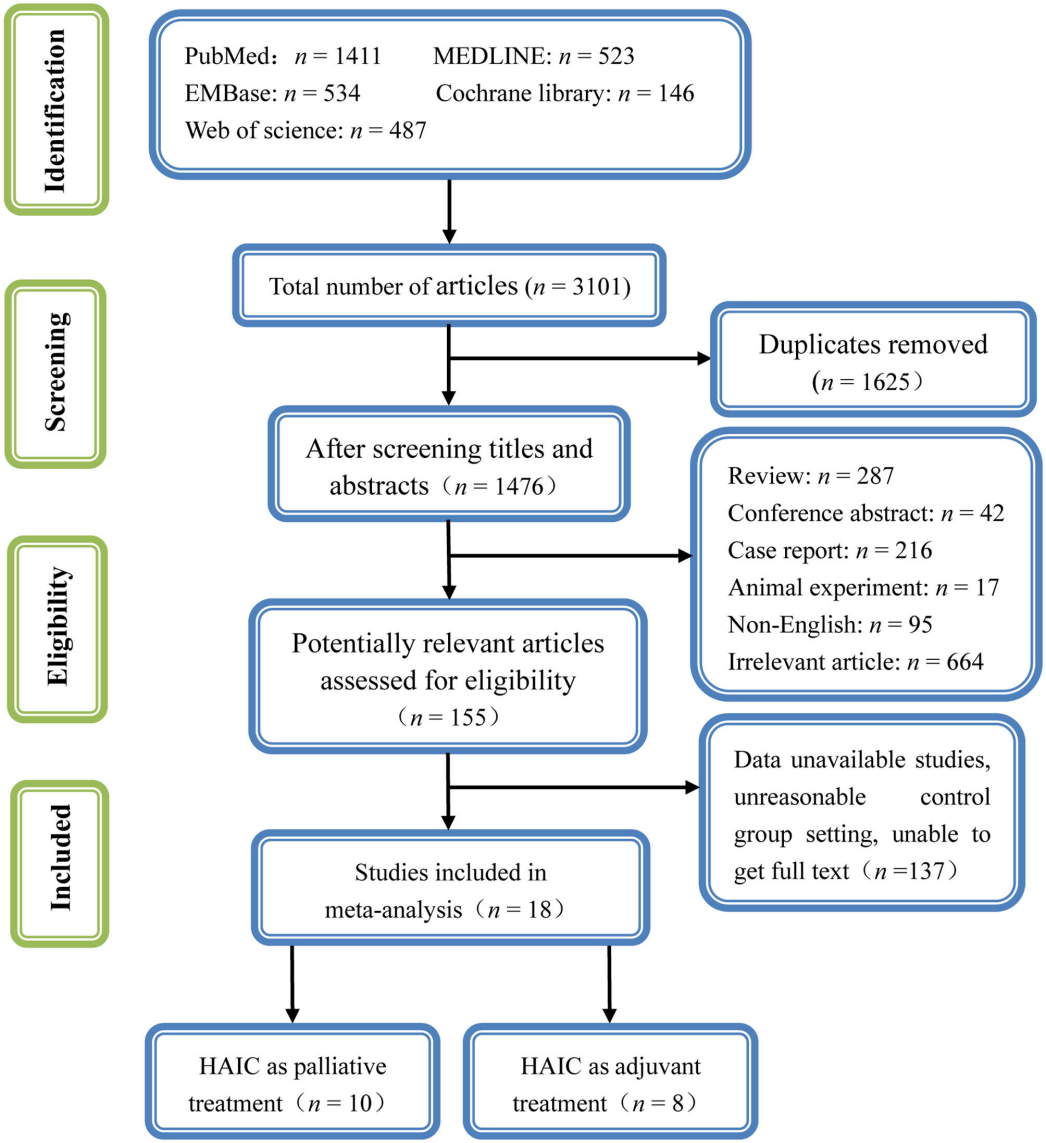
Flowchart presenting the selection process of studies.

A total of 2,197 potentially relevant articles were retrieved using the search strategy described above. Among these, 918 were duplicates. A total of 1,168 articles were excluded by screening the titles and abstracts as they were reviews, letters, comments, not in English, case reports, or laboratory studies, leaving 111 articles. A further 93 articles were excluded by examining the abstracts or full-texts. Finally, 18 studies (10–27) met the inclusion criteria and were included in the present meta-analysis. The detailed flowchart of the selection process for eligible studies is shown in Figure 2.

### Study Characteristics

A total of 18 studies involving 1,766 participants were included in the present meta-analysis. Among these, 922 were allocated to the HAIC group, and 844 patients were allocated to the SC group. 10 studies (10–13, 15, 16, 19, 21–23) applied HAIC as a palliative treatment in patients with unresectable colorectal liver metastases, and 8 studies (14, 17, 18, 20, 24–27) used it as an adjuvant treatment in patients with curative resection of liver metastases. These studies were published between 1987 and 2019. In nine studies (50%), FUDR was the only drug used for HAIC (10–16, 22), 5-FU alone was used in two studies (20, 25), and the remaining seven studies (17, 18, 21, 23, 24, 26, 27) used a combination of drugs for HAIC. In the trial with three groups, either FUDR or 5-FU plus leucovorin was administered through HAIC. The regimens adopted in the SC group were, FUDR alone in three trials (10–12), 5-FU alone in four trials (13, 14, 20, 25), UFT alone in one study, and in the other 10 trials (15–17, 19, 21–24, 26, 27), a combination of regimens were used. With regards to the outcomes, 17 trials reported OS (10–23, 25–27), 11 trials reported RRs (10–15, 19, 21–23), 7 trials reported PFS (13, 17, 19, 21–23, 27). The characteristics of the included studies are presented in Table 1.

**Table 1:** Characteristics of included studies.

**Study ID**	**Year**	**Region**	**Ages**		**Sample size**	**Regimens of treatment group**	**Regimens of control group**	**Outcomes**
			**Combination group**	**SC group**				
Chang et al.	1987	America	57 (37–77)	61 (37–70)	64	HAIC: FUDR	SC: FUDR	OS, RRs
Kemeny et al.	1987	America	60 (36–76)	61 (37–75)	99	HAIC: FUDR	SC: FUDR	OS, RRs
Hohn et al.	1989	America	61.2 (34–79)	61.8 (36–79)	115	HAIC: FUDR	SC: FUDR	OS, RRs
Martin et al.	1990	America	NR	NR	69	HAIC: FUDR	SC: 5-FU	OS, RRs, PFS
Wagman et al.	1990	America	57.8 (37–73)	67 (54–76)	41	HAIC: FUDR	SC: 5-FU	OS, RRs
Rougier et al.	1992	France	59 ± 8	61 ± 10	163	HAIC: FUDR	SC: 5-FU/BSC	OS, RRs
Allen-Mersh et al.	1994	England	55 ± 10	59 ± 8	100	HAIC: FUDR	SC: 5-FU/BSC	OS
Kemeny et al.	1999	America	59 (28–79)	59 (30–77)	156	HAIC: FUDR+DXM+LV+5-FU	SC: LV+5-FU	OS, PFS
Kusunoki et al.	2000	Japan	60.0 (25–71)	55.5 (33–75)	58	HAIC: 5-FU+UFT	SC: UFT	OS
Lorenz et al.	2000	Germany	60.5 (33–78)	62 (37–80)	168	HAIC: FUDR/5-FU+LV	SC: 5-FU/LV	OS, RRs, PFS
Tono et al.	2000	Japan	59.0 ± 5.8	61.9 ± 5.0	19	HAIC: 5-FU	SC: 5-FU	OS
Kerr et al.	2003	England	63	62	290	HAIC: 5-FU+LV	SC: 5-FU+LV	OS, RRs, PFS
Kemeny et al.	2006	America	57 (21–81)	61 (35–86)	135	HAIC: FUDR	SC: 5-FU+LV	OS, RRs, PFS
Fiorentini et al.	2012	Italy	64 (44–74)	63 (42–73)	74	HAIC: CPT-11	SC: CPT-11+5-FU+LV	OS, RRs, PFS
Li et al.	2016	China	78 (75–80)	77.5 (75–82)	51	HAIC: FUDR+CAP	SC: CAP	RRs
Kusano et al.	2017	Japan	63.0 (40–80)	62.5 (46–80)	88	HAIC:5-FU	SC: 5-FU	OS
Kusano et al.	2018	Japan	62.4 (45–78)	64.2 (32–77)	44	HAIC: 5-FU+LV+UFT	SC: LV+UFT	OS
Ghiringhelli et al.	2019	France	65.6(44.5–82.4)	54.7 (39.9–81.9)	27	HAIC: raltitrexed+OXA	SC: AP/MMC/regorafenib	OS, PFS

*HAIC, hepatic arterial infusion chemotherapy; SC, systemic chemotherapy; 5-Fu, fluorouracil; FUDR, floxuridine; LV, leucovorin; UFT, uracil tegafur; OXA, oxaliplatin; CAP, capecitabine; MMC, mitomycin C; CPT-11, irinotecan; DXM, dexamethasone; BSC, best supportive care; NR, not reported*.

### Study Quality Assessment

**Figure 3:**
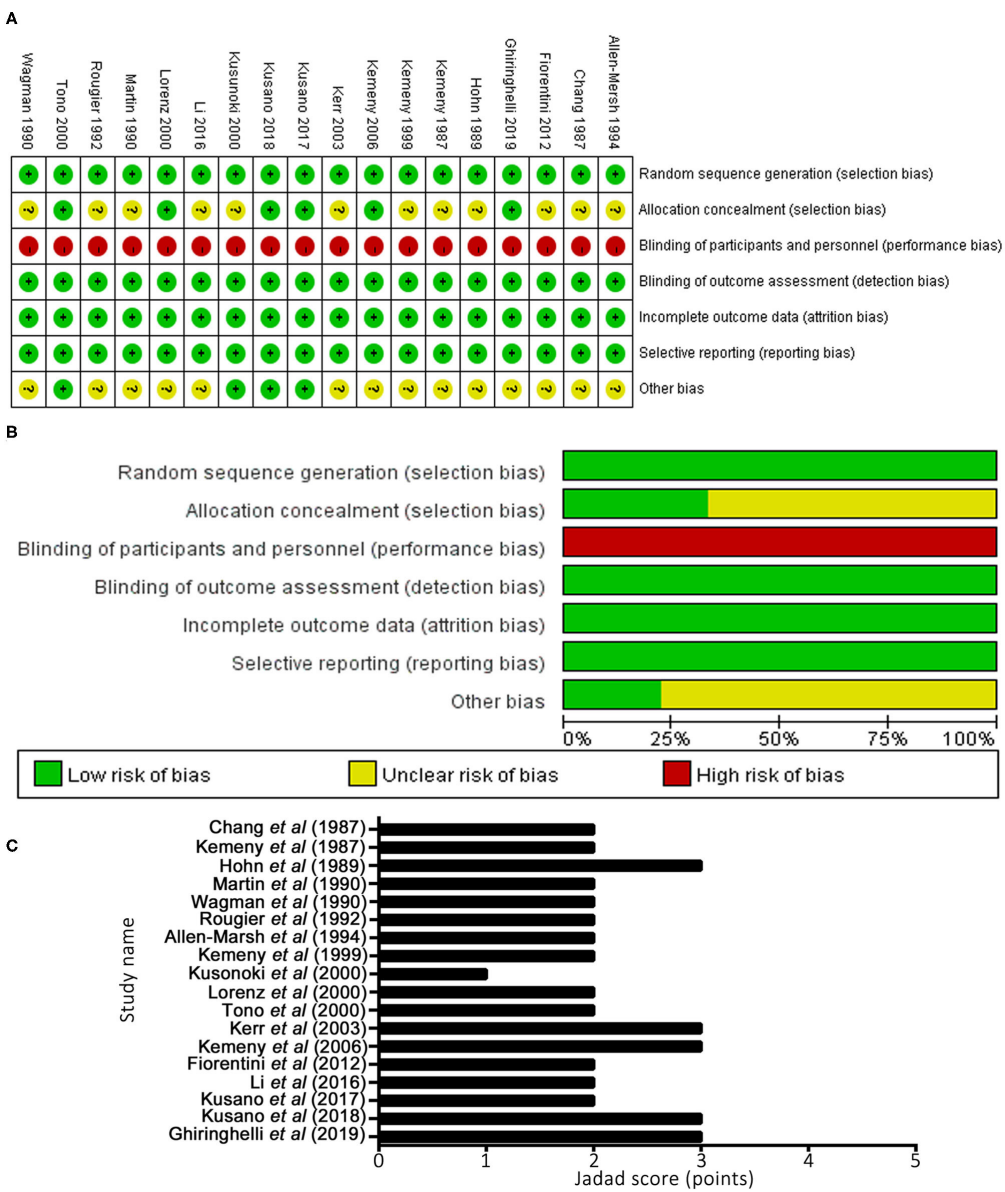
Methodological quality graph and summary of the included studies: **(A)** Risk of bias summary; **(B)** Risk of bias graph; **(C)** Jadad scoring system.

Methodological quality graphs and a summary of the included studies are presented in Figures 3A,B. The generation of randomized sequence was identified adequately in all trials. Appropriate allocation concealment was missing in several trails. None of studies had robust double blinding procedures. The results of quality assessment, based on Jadad's scale, are presented in Figure 3C. The scores of included studies ranged between 1 and 3 points (mean, 2.22). A total of five studies (12, 21, 22, 26, 27) reported sequence generation and ensured random allocation. One trial (18) scored 1 point overall due to inappropriate allocation concealment. All included studies reported withdrawal/dropout rates. Therefore, five studies (12, 21, 22, 26, 27) out of 18 studies were considered high quality (≥3 points).

### OS

**Figure 4:**
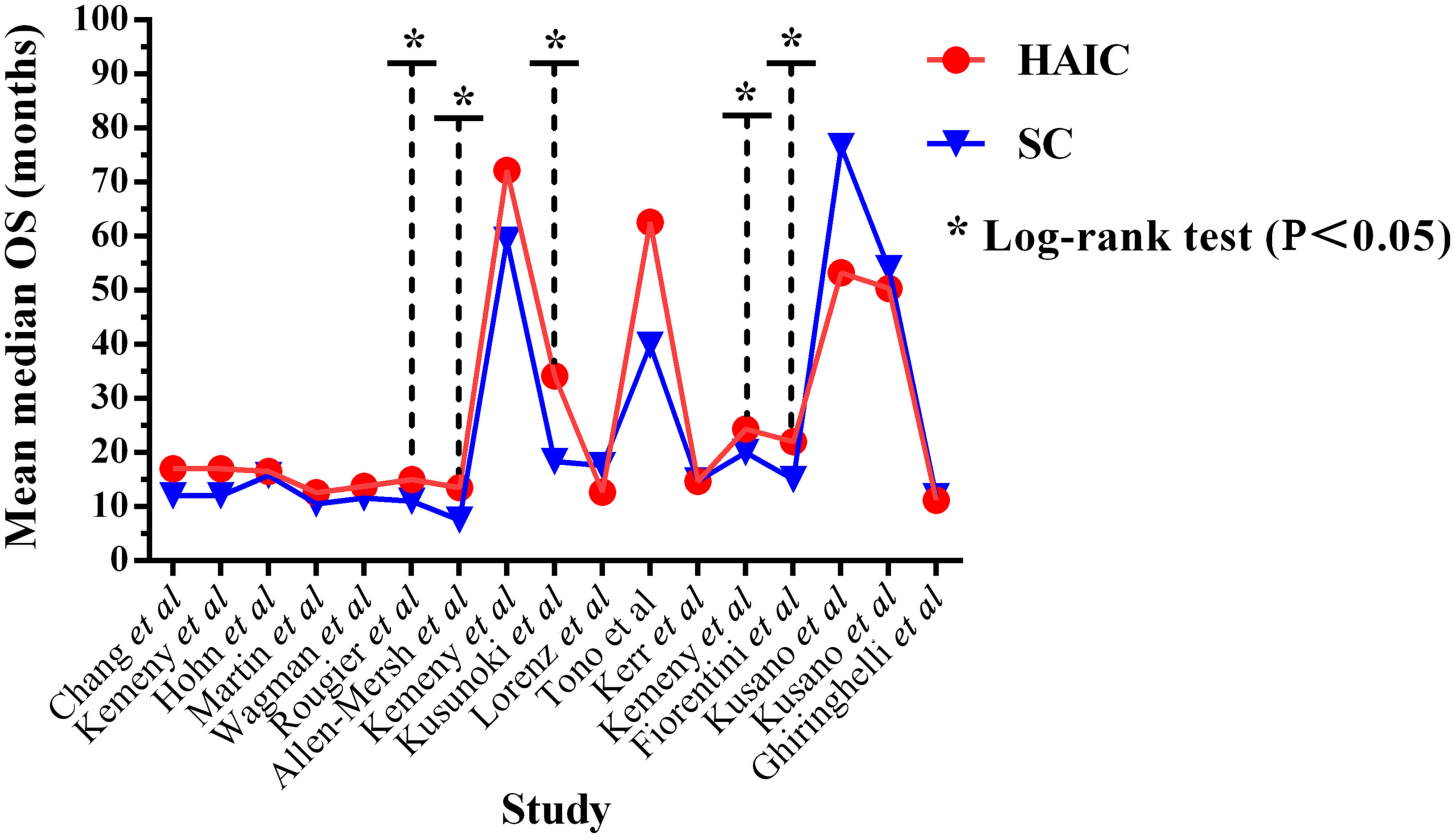
Mean median overall survival time.

OS outcomes were analyzed in 17 trials (10–23, 25–27), including a total of 1,715 patients. Five trials (15, 16, 18, 22, 23) out of 17 reported significantly improved median OS in the HAIC group (*P* < 0.05). The median OS time ranged between 11.2 and 72.2 months (mean, 27.24 months) in the HAIC group, and 11.0–76.6 months (mean, 23.99 months) in the SC group (Figure 4). Ten studies (10–13, 15, 16, 19, 21–23) applied HAIC as a palliative treatment in patients with unresectable colorectal liver metastases, and 7 studies (14, 17, 18, 20, 25–27) used it as an adjuvant treatment in patients with curative resection of liver metastases.

**Figure 5:**
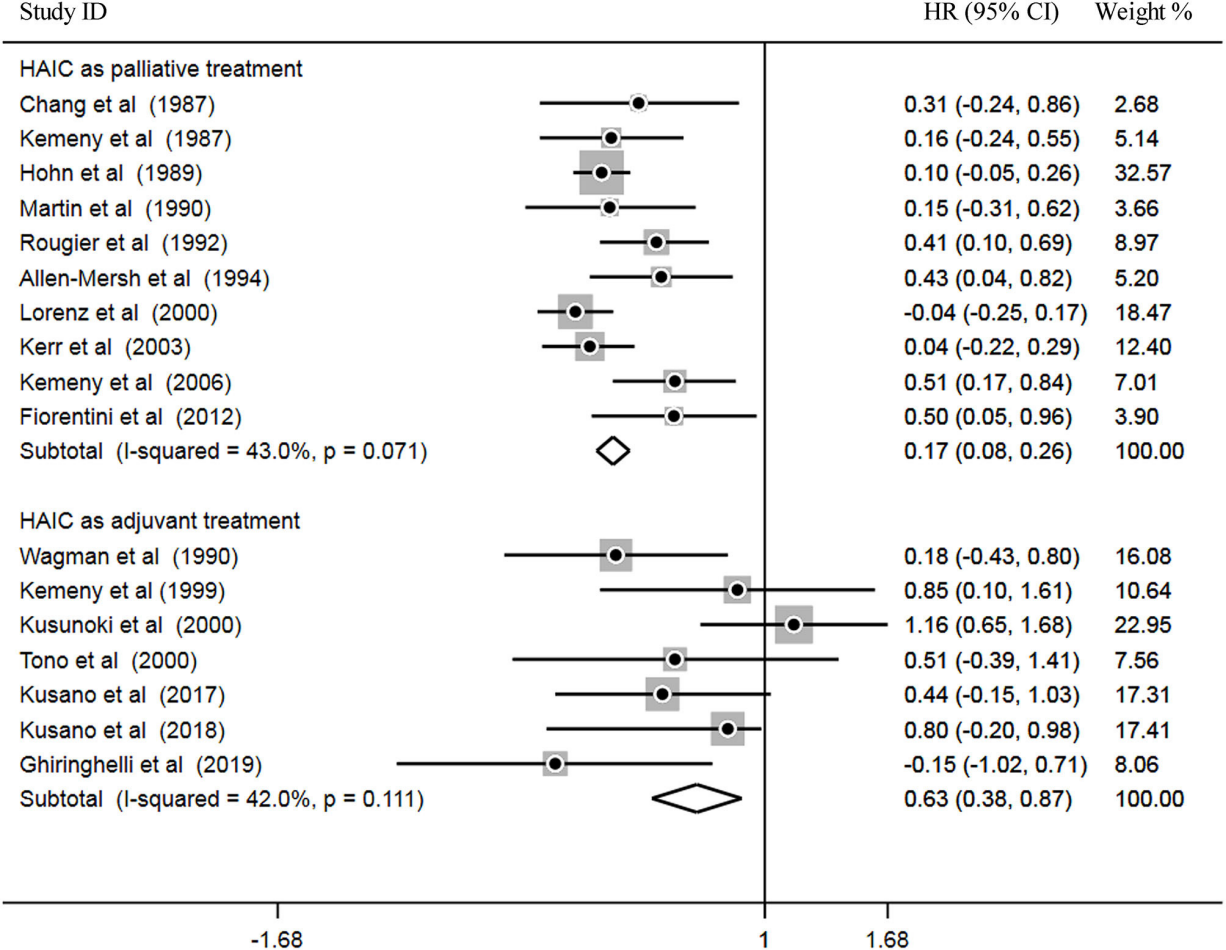
Forest plot of hazard ratios (HR) for OS.

Subsequently, heterogeneity was examined prior to pooled analysis. Test results revealed there were no significant heterogeneity across 10 palliative studies (*P* = 0.071, I^2^ = 43.0%) and 7 adjuvant studies (*P* = 0.111, I^2^ = 42.0%). Thus, a fixed effects model was applied for the pooled analysis. In the pooled meta-analysis, OS was significantly increased in the HAIC as palliative treatment group compared with patients in the SC group (Z = 3.66, *P* = 0.000; HR, 0.17; 95% CI, 0.08–0.26). Furthermore, OS was significantly increased in the HAIC as adjuvant treatment group compared with SC group (Z = 3.99, *P* = 0.000; HR, 0.63; 95% CI, 0.38–0.87). These results showed that HAIC was an effective treatment that increased OS. Pooled analysis results are presented in Figure 5.

### Tumor RRs

In total, 11 trials out of 18 reported RRs, and all 1,022 patients in these studies were included for pooled analysis. Among these, nine studies (2–5, 7, 9, 14, 18, 28) applied HAIC as a palliative treatment in patients with unresectable colorectal liver metastases, and two studies (14, 24) used it as an adjuvant treatment in patients with curative resection of liver metastases.

**Figure 6:**
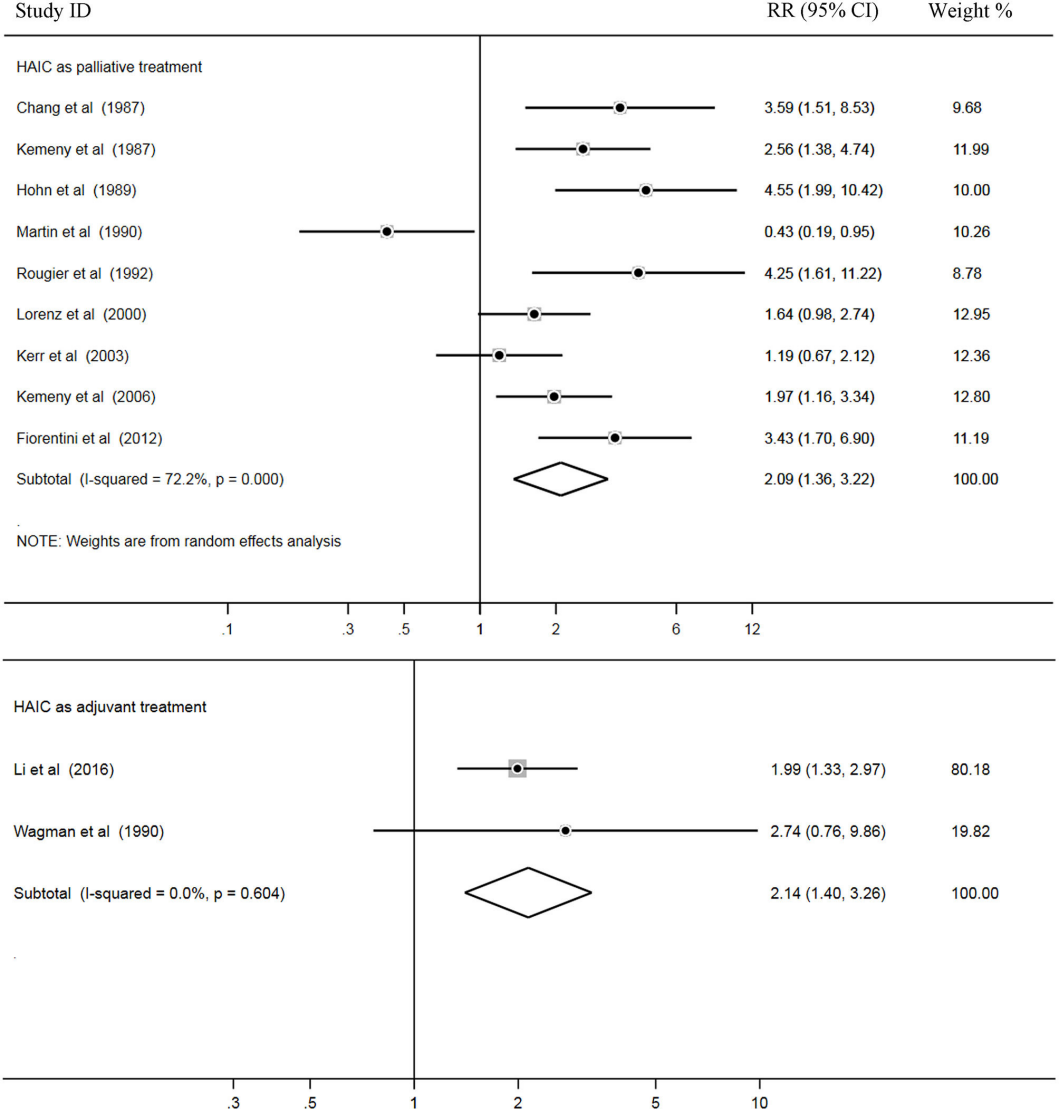
Forest plot of relative risk (RR) for tumor response rates.

Heterogeneity among the studies was also examined. The results showed that there was statistical heterogeneity among nine palliative studies (*P* = 0.000, I^2^ = 72.2%). Thus, a random effects model was used for pooled analysis. In addition, there were no significant heterogeneity across two adjuvant studies (*P* = 0.604, I^2^ = 0.0%) and a fixed effects model was applied for the pooled analysis. Pooled data demonstrated higher rates of RRs in the HAIC as palliative treatment group compared with the SC group (Z = 3.36, *P* = 0.001; RR = 2.09; 95% CI, 1.36–3.22). In addition, RRs was significantly increased in the HAIC as adjuvant treatment group compared with SC group (Z = 3.53, *P* = 0.000; RR = 2.14; 95% CI,1.40–3.26). Pooled analysis is presented in Figure 6.

### PFS

**Figure 7:**
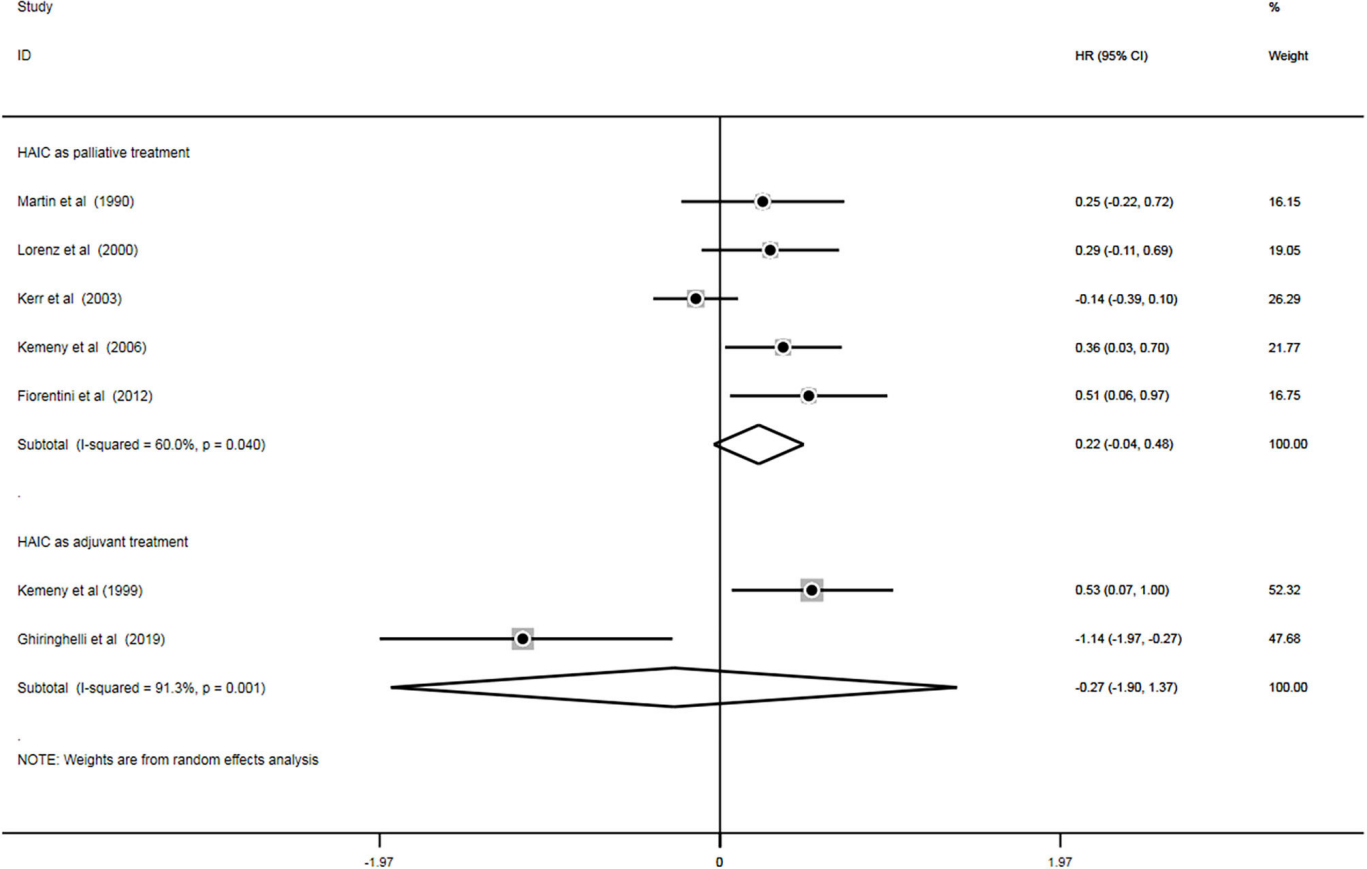
Forest plot of hazard ratios (HR) for progression-free survival (PFS).

A total of seven trials (11, 18, 20, 22–24, 28) out of 18 analyzed the impact of treatment on PFS (*n* = 924). Of these, five studies (13, 19, 21–23) applied HAIC as a palliative treatment and two studies (17, 27) used it as an adjuvant treatment. Heterogeneity was examined prior to pooled analysis. Test results revealed there were significant heterogeneity across 5 palliative studies (*P* = 0.040, I^2^ = 60.0%) and 2 adjuvant studies (*P* = 0.001, I^2^ = 91.3%). Thus, a random effects model was applied for the pooled analysis. In the pooled meta-analysis, PFS were neither significantly increased in the HAIC as palliative treatment (Z = 1.69, *P* = 0.091; HR, 0.22; 95% CI, −0.04–0.48) nor as adjuvant treatment (Z = 0.32, *P* = 0.750; HR, −0.27; 95% CI, −1.90–1.37). Meta-analysis of the pooled data demonstrated that PFS outcomes did not differ between the HAIC and the SC group. Pooled analysis results are presented in Figure 7.

### Sensitivity Analysis

**Figure 8:**
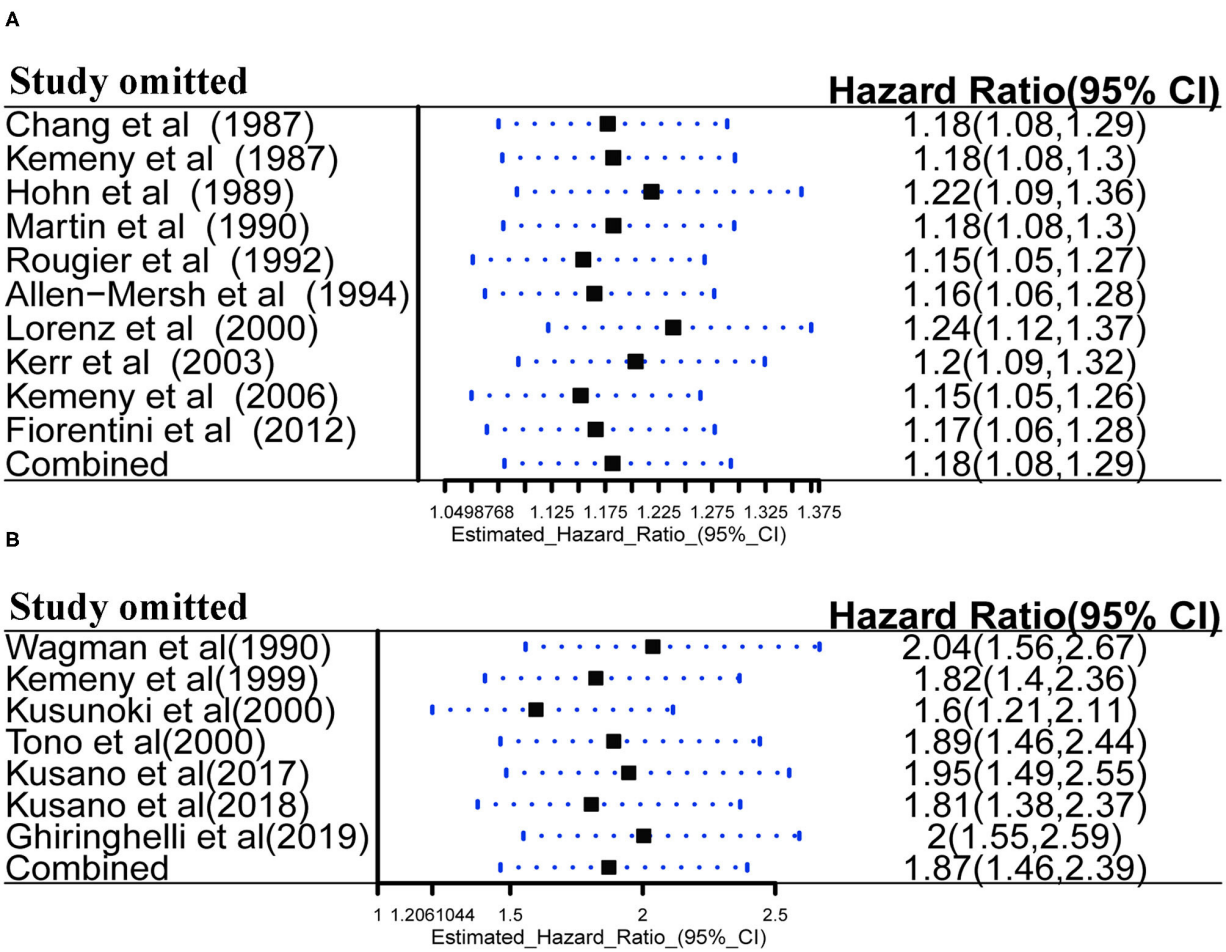
**(A)** Sensitivity analysis of HAIC as palliative treatment for OS; **(B)** Sensitivity analysis of HAIC as adjuvant treatment for OS.

Robustness of OS was further confirmed by sensitivity analysis in palliative treatment group (Figure 8A) and adjuvant treatment group (Figure 8B). Sensitivity analysis was performed using a leave-one-out at a time procedure, and the results showed that exclusion of any individual study did not significantly skew the pooled effect (*P* < 0.05), indicating that the results of pooled analysis for OS were robust to some extent.

### Publication Bias

**Figure 9:**
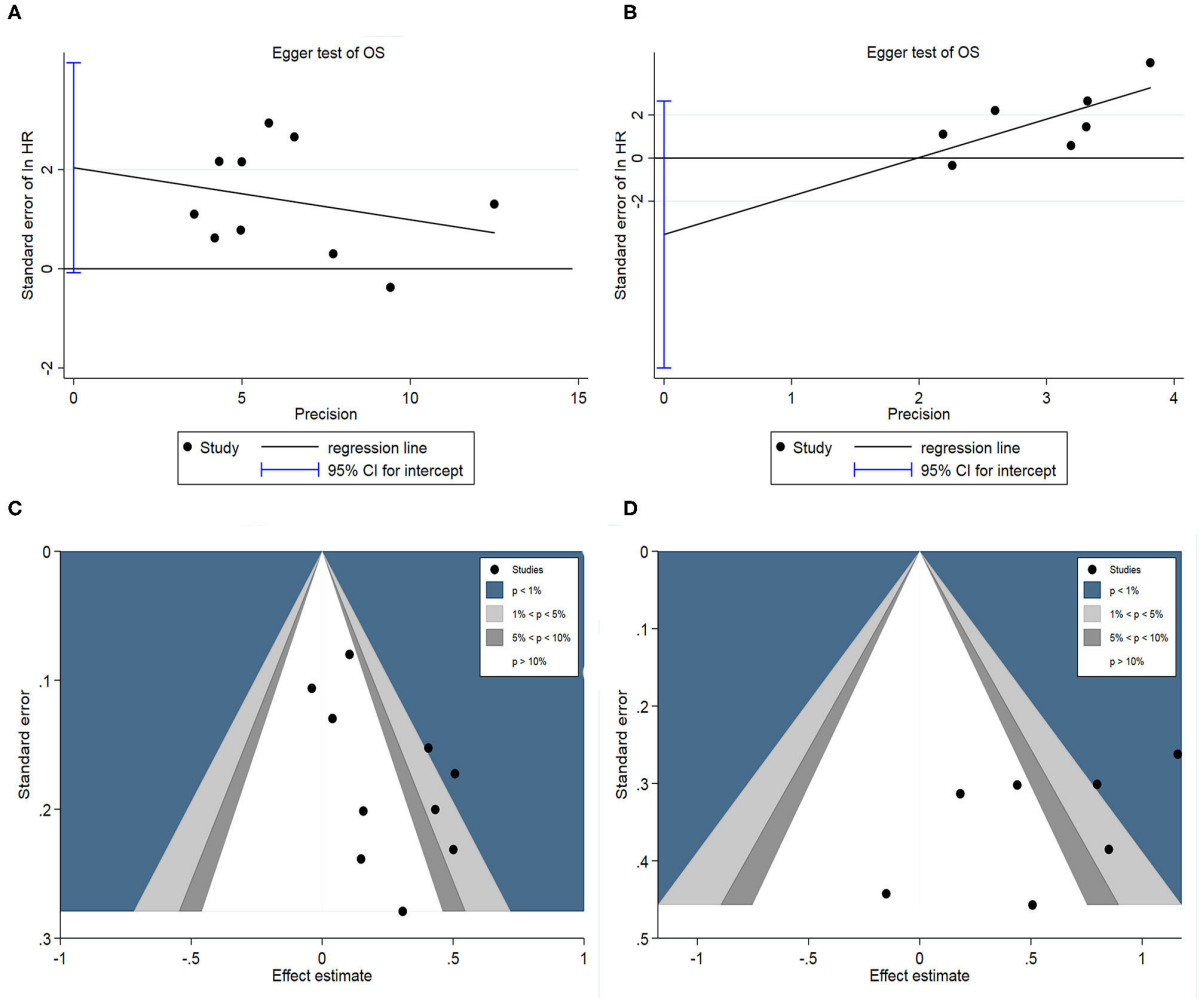
**(A)** Egger's funnel plot of HAIC as palliative treatment for OS; **(B)** Egger's funnel plot of HAIC as adjuvant treatment for OS; **(C)** Contour-enhanced funnel plot for HAIC as palliative treatment; **(D)** Contour-enhanced funnel plot for HAIC as adjuvant treatment.

Egger's test and contour-enhanced funnel plot were used to assess potential publication bias. Firstly, Egger's test was used to assess potential publication bias in the pooled OS as the results are quantitative. Egger's test showed there were no significant publication bias in HAIC as palliative treatment group (*P* = 0.057; Figure 9A) and adjuvant treatment group (*P* = 0.201; Figure 9B) in this meta-analysis. Subsequently, a contour-enhanced funnel plot, which added conventional milestones in levels of statistical significance (*P* < 0.1, *P* < 0.05, *P* < 0.01) to funnel plots, was utilized to distinguish detailed reasons of publication bias. Results indicated several missing studies were in areas of higher statistical significance (*P* < 0.01, Figures 9C,D), highlighting that the potential reason of the asymmetry may be due to factors other than publication bias. Finally, the original research was traced again, speculating that lower methodology quality (such as non-double-blinded design, unsatisfactory calculation of power and small sample sizes) may account for the bias. These limitations may undermine the reliability of the results.

### Meta-Regression Analysis

Meta-regression was performed to assess the effects of any underlying confounding factors on the pooled effect, and to identify potential sources of heterogeneity in the OS. The following covariates were considered as potential factors: (i) Different treatment regimens, FUDR alone vs. non-FUDR; (ii) sample size, *n* ≥ 100 vs. *n* < 100; and (iii) methodology quality assessment, high quality vs. low quality. Overall, univariate analysis showed all these three covariates did not exert any significant influence on the association between HAIC as a palliative or as adjuvant treatment and OS outcome (*P* > 0.05, Table 2). Subsequently, multivariate meta-regression was used to assess the effect of various covariates on the pooled effect of OS. The results revealed that all these three variables did not affect the relationship between HAIC and OS (*P* = 0.43) and heterogeneity was not observed based on this model. The results are shown in Table 2.

**Table 2:** Meta-regression analysis of overall survival (OS).

**Covariates**	**HAIC as palliative treatment (10 studies)**				**HAIC as adjuvant treatment (7 studies)**			
	**Exponentiated coefficient**	**95% CI^**&**^**	***P***	**Tau^**2**^**	**Exponentiated coefficient**	**95% CI^**&**^**	***P***	**Tau^**2**^**
**Univariate analysis**								
Treatment regimens	0.25	−0.29 to 0.79	0.323	0.026	0.49	−0.65 to 1.63	0.32	0.068
Sample size	0.16	−0.62 to 0.94	0.646	0.025	0.30	−1.09 to 1.69	0.60	0.108
Methodology quality	0.30	−0.21 to 0.82	0.212	0.026	0.87	−0.55 to 2.29	0.18	0.114
**Multivariate analysis**								
Treatment regimens	0.01	−0.55 to 0.53	0.95	NA	0.56	−1.28 to 2.34	0.40	NA
Sample size	0.09	−0.46 to 0.64	0.71	NA	0.11	−1.86 to 2.08	0.87	NA
Methodology quality	0.08	−0.55 to 0.38	0.67	NA	0.33	−1.85 to 1.18	0.54	NA
Omnibus test for moderators			0.64	0.04			0.72	0.149

& *CI, Confidence Intervals; NA, Not Applicable*.

## Discussion

To the best of our knowledge, the present meta-analysis is the first study to show the potentially positive benefits of HAIC in improving OS among CRLM patients compared with SC. This integrated analysis, which included 18 prospective RCTs with 1,766 participants, demonstrated that CRLM patients treated with HAIC had significantly higher OS rates compared with those treated with SC. HAIC as a palliative treatment in patients with unresectable colorectal liver metastases and as an adjuvant treatment in patients with curative resection of liver metastases were likely to prolong the OS time of CRLM patients. The rates of complete and partial RRs also increased significantly in the HAIC group compared with the SC group. However, PFS did not differ significantly between the two groups. These data demonstrate that HAIC may be an effective intervention for the treatment of CRLM, particularly in improving OS and RRs. However, pooled data demonstrated that PFS outcomes were not different between the HAIC and the SC group. This result may be interpreted as both HAIC and SC may effectively reduce progression or recrudescence of tumors.

HAIC is a mode of chemotherapeutic drug administration. HAIC, as a locoregional therapy, has significant advantages in terms of pathological RRs with a ≥ 6-fold increase in effective dose in CRLM patients (29). Owing to anatomical features, liver metastases are formed primarily from the blood supply from the hepatic artery, whereas normal liver tissue is primarily perfused by the portal vein; thus, a significantly higher local concentration of chemotherapeutic drugs can be administered *via* HAIC (28, 30). Furthermore, the metabolic processes of chemotherapeutic drugs *in vivo*, including absorption, distribution, metabolism, and excretion also affect clinical efficacy. FUDR, 5-Fu, oxaliplatin, or molecular targeting agents (such as bevacizumab and cetuximab) administered *via* HAIC have a short half-life and are metabolized primarily in the liver, thus allowing extremely low drug concentrations in the peripheral blood and reducing the effect of first-pass hepatic metabolism. As a result, HAIC is suitable for administration of effective higher doses of drugs directly to tumors, and thus lowers the risk of adverse systemic events (36).

The higher RRs of HAIC may underlie the improved OS rates, hypothetically. Favorable outcomes, such as prolonging survival and reducing tumor progression, were reported in previous studies. Rougier et al. (15) described a significant improvement in survival in patients treated with HAIC compared with SC with regards to the 1-year survival rates (64 vs. 44%) and 2-year rates (23 vs. 13%). The median survival times were improved in the HAIC group compared with the SC group (15 vs. 11 months), respectively. Allen-Mersh et al. (16) demonstrated improved OS in the HAIC group compared with the SC group (median survival time 405 vs. 226 days). Additionally, the quality of life was maintained even with long-term treatment. Another two trials also showed patients with CRLM had longer OS times when treated with HAIC (18, 22). In recent years, novel chemotherapeutic drugs, such as irinotecan, oxaliplatin, bevacizumab, and cetuximab are widely administrated through HAIC in clinical practice, contributing to longer survival times of >20 months in patients with CRLM (37, 38). These data suggest the potential of HAIC for the management of CRLM. However, based on available evidence currently, HAIC used as an adjuvant or as a palliative treatment may lead to different results. According to the conclusions from original studies (21, 23, 28, 29), HAIC as adjuvant treatment after curative liver resection (R0/R1) in CRLM patients did not show survival benefits. Thus, the conclusion from this study deduced that HAIC could serve as a palliative treatment for patients with CRLM and could beneficial to a longer survival time.

This integrated analysis provides evidence suggesting that HAIC is effective at controlling CRLM. However, potential limitations should be noted when accepting the conclusions of the present study. First, side-effects related to HAIC have been reported in several studies. Adverse effects may be technical or harmful when the pump and catheter are placed. Complications of pump placement, such as catheter-connected events, including hepatic artery occlusion, thrombosis and catheter-related infection, have garnered increasing attention, even though the rates were <7% (39, 40). Gastrointestinal symptoms, such as hyperbilirubinemia, biliary sclerosis, nausea, diarrhea, vomit, and stomatitis were observed in 25–35% of patients treated with HAIC (41, 42). Secondly, there was notable heterogeneity and bias between studies, which should be taken into consideration. Variations in the duration of administration of the chemotherapeutic drugs used in the HAIC and SC groups, inconsistent baseline data, such as the number of metastases, tumor size and location may result in heterogeneity. In addition, the improvement in OS from HAIC should also be assessed based on whether the liver metastases are resected. Finally, the methodological limitations should be acknowledged. None of the included studies had robust double blinding procedures, allocation concealment was missing in several studies, and small sample sizes may have resulted in selection and performance bias. All these factors may result in instability in the present analysis. Thus, more prospective studies with larger samples sizes, long-term survival time evaluation and standardized protocols are required to accurately determine the role of HAIC in controlling colorectal liver metastases.

## Data Availability Statement

Publicly available datasets were analyzed in this study. This data can be found here: https://pubmed.ncbi.nlm.nih.gov/, https://www.crd.york.ac.uk/prospero/.

## Author Contributions

YZ, KW, and TY performed the search and drafted the manuscript. YC and WL performed the data extraction and analyzed the data. YZ and TY designed the study and amended the original draft. XY and TX provided the clinical imaging data of the patients and equally contributed to the conception of the study. All authors contributed to the article and approved the submitted version.

## Conflict of Interest

The authors declare that the research was conducted in the absence of any commercial or financial relationships that could be construed as a potential conflict of interest.
